# A new technique of intramedullary elastic reduction of the “de-sharpened” Kirschner wire for the treatment of Gartland type III posterolateral displaced supracondylar fracture of the humerus in children

**DOI:** 10.1186/s40001-024-01671-4

**Published:** 2024-01-30

**Authors:** Yudong Lin, Zhongtuo Hua, Cheng Zhou, Saiwen Chen, Xiwei Sun, Fang Liu, Ge Meng, Sicheng Zhang, Jun Sun

**Affiliations:** 1 Anhui Provincial Children’s Hospital, Hefei, China; 2 The Fifth Clinical College of Anhui Medical University, Hefei, China

**Keywords:** Supracondylar fracture of the humerus, Kirschner wire, Operation

## Abstract

**Objective:**

To compare the clinical effects of intramedullary elastic reduction of the “de-sharpened Kirschner wire and traditional three-dimensional manipulation in the treatment of Gartland type III posterolateral supracondylar fracture of the humerus in children.

**Methods:**

A retrospective cohort analysis was made on 106 cases of Gartland type III posterolateral supracondylar fracture of the humerus treated in the Department of Orthopaedics of a Children’s Hospital from March 2020 to March 2022. According to different surgical technology, the patients were divided into two groups: intramedullary elastic reduction of the de-sharpened Kirschner wire group (experimental group, *n* = 50) and traditional three-dimensional manipulation group (control group, *n* = 56). The surgical operating time, intraoperative fluoroscopy times, postoperative Baumann angle changes, postoperative elbow function Flynn score, and complications were collected and compared between the two groups.

**Results:**

All the enrolled cases underwent surgery successfully and were followed-up at least 6 months. The surgical operating time of the experimental group was 32.88 ± 3.69 min and that of the control group was 45.56 ± 10.13 min, and the difference was statistically significant (*P* < 0.05). The intraoperative fluoroscopy times were 20.62 ± 5.41 times in the experimental group and 32.48 ± 8.20 times in the control group (*P* < 0.05). The change of Baumann angle in the experimental group after operation was 2.3 ± 1.3 and that in the control group was 6.0 ± 2.1 (*P* < 0.5). Elbow joint Flynn scoring standard to evaluate the curative effect: the excellent and good rate was 98.00% (49/50) in the experimental group and 92.86% (52/56) in the control group (*P* > 0.5). There were no complications such as osteomyelitis, compartment syndrome, iatrogenic vascular and nerve injury, and myositis ossificans in either group.

**Conclusions:**

Good functional outcome can be obtained with both intramedullary elastic reduction of the de-sharpened Kirschner wire and traditional three-dimensional manipulation for Gartland type III posterolateral displaced supracondylar fracture of the humerus in children; however, the former does not need repeated manipulation, and the operation time is shorter, the number of intraoperative fluoroscopy is less, and the recovery of the Baumann angle is better.

## Introduction

Supracondylar fracture of the humerus is the most common elbow fracture in children. In the classification of injury mechanism, extension fracture is common, and Gartland type III fracture should be treated by closed reduction and percutaneous Kirschner wire fixation [1]. On the basis of the traditional Gartland classification, Wilkins [2] divided type III fractures into type IIIa (posteromedial displacement) and type IIIb (posterolateral displacement). In the treatment of Gartland type III fractures, three-dimensional reduction [3], followed by percutaneous Kirschner wire internal fixation is usually used to maintain the stability of the fracture. In the posterolateral supracondylar fracture of the humerus, most of the medial periosteum is completely broken and the lateral periosteum is also very weak. If the operator exerts too much force in supination reduction, it is easy to break the medial and lateral periosteum at the same time. Further, in supracondylar fracture of the humerus with severe posterolateral displacement, the medial side of the proximal end of the fracture is spiked and rotated forward at the same time, and the broken end can easily be inserted into muscle. When traditional three-dimensional reconstruction is used, it is sometimes difficult to correct coronal and rotational deformities, and it is difficult to maintain fracture stability in extreme flexion of the elbow joint. In this case, it becomes necessary to try manual reduction and C-arm fluoroscopy multiple times. This may lead to the aggravation of the surrounding soft tissue injury that may result in the dysfunction of elbow joint [4]. Repeated reset and fluoroscopy also greatly increase the risk of radiation exposure to medical staff. In recent years, the authors have used the technique of intramedullary elastic reduction of the “de-sharpened” Kirschner wire in the treatment of posterolateral displaced supracondylar fracture of the humerus in children. The treatment is accurate and effective, and the details are reported below.

## Materials and methods

In all, 106 patients (58 male and 48 female, age range: 4–12 years) with posterolateral displaced supracondylar fracture of the humerus (38 right sided and 68 left sided) treated in the Department of Orthopaedics from March 2020 to March 2022 were analyzed. The time from injury to surgery ranged between 12 h and 4 days. All patients were treated with simple traction reduction and plaster fixation of the upper limb as a temporary treatment. The inclusion criteria were as follows: (i) no history of limb fracture before injury; (ii) new fracture; (iii) significantly displaced posterolateral Gartland III type fracture (no bone cortex contact); and (iv) provision of parental consent to participate in the study. The exclusion criteria were as follows: (i) open fracture; (ii) pathological fracture; (iii) other co-existing fractures; (iv) underlying vascular and nerve injury; and (v) congenital upper arm deformity. Surgeon’s preferences determined methods of operation. According to different surgical methods, the patients were divided into two groups: intramedullary elastic reduction of the de-sharpened Kirschner wire group (experimental group, *n* = 50) and the traditional three-dimensional reduction group (control group, *n* = 56). In two groups were similar respect to sex, gender, side, BMI, injured cause and the time from injury to operation (*P* > 0.05) (Table 1). All patients signed informed consent. This study was approved by the Medical Ethics Committee of our institution (EYLL-2022-043).

**Table 1:** Comparison of patients’ general information between two groups of Gartland type III posterolateral displaced supracondylar fracture of the humerus in children (*n*)

Groups	*n*	Gender		Age (year, $\overline{x}$ ± *s*)	Cause		Affected side		BMI	Injury time (h, $\overline{x}$ ± *s*)
		Male	Female		Falling	Traffic	Right	Left		
Study group	50	28	22	8.5 ± 2.6	32	18	20	30	17.4 ± 2.6	29.5 ± 7.9
Control group	56	30	26	8.6 ± 2.1	39	17	18	38	17.3 ± 2.6	31.5 ± 7.5
${x}^{2}$/*t* value		0.06		− 0.73	0.38		0.71		0.37	− 1.34
P value		0.802		0.469	0.537		0.400		0.713	0.183

Study group were treated with “de-sharpened” intramedullary elastic reduction with Kirschner wire, Control group were treated with traditional three-dimensional manipulation

### Surgical technique

All patients lay on the operating bed and received general anesthesia and brachial plexus block anesthesia. The C-arm receiver was placed at the level of the patient's shoulder and parallel to the operating table. At this point, the injured elbow can be completely placed on the C-arm receiver, and the C-arm receiver can be used instead of the operating table. During the operation, we used a semisterile technique [5] and covered the patient's hands with aseptic gloves. First, the assistant stood on the opposite side of the operator holding the proximal humerus, and the operator held the forearm to resist traction and correct the overlapping insertion and displacement of the fracture. If the “pucker” sign of the anterior medial part of the elbow could be seen, the proximal medial fracture tip of the fracture was withdrawn from the humerus muscle by gradual extrusion [6].

#### Experimental group

**Fig. 1:**
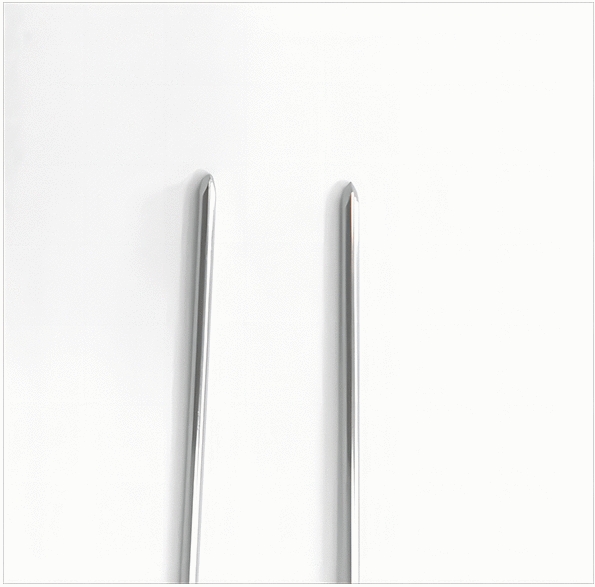
On the left is a 2.0mm 'de-sharpened' Kirschner wire, with the Kirschner wire tip sharpened using a file. On the right is a standard-tipped Kirschner wire

**Fig. 2:**
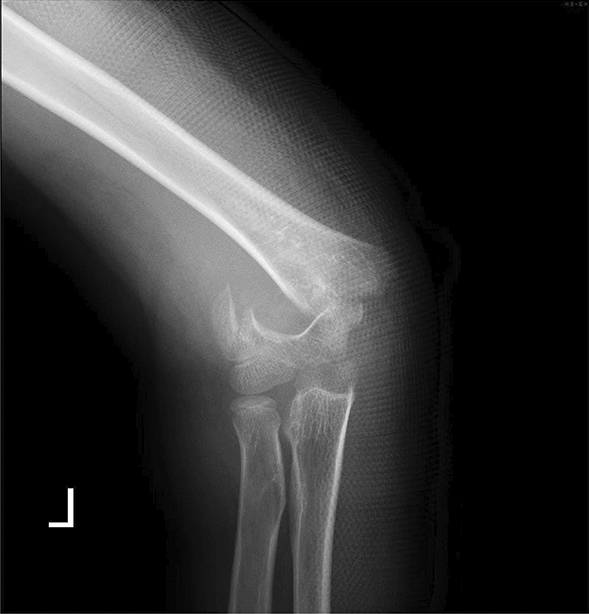
Post-injury X-ray in the anterior-posterior view shows a fracture at the humeral condyle, with significant lateral displacement of the distal bone fragment

**Fig. 3:**
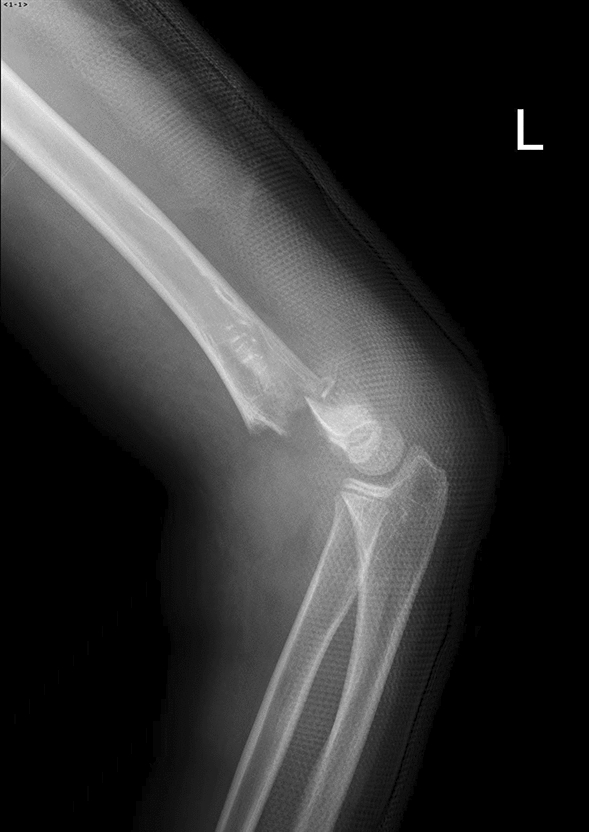
Post-injury X-ray in the lateral view shows a fracture at the humeral condyle, with complete backward displacement of the distal bone fragment

**Fig. 4:**
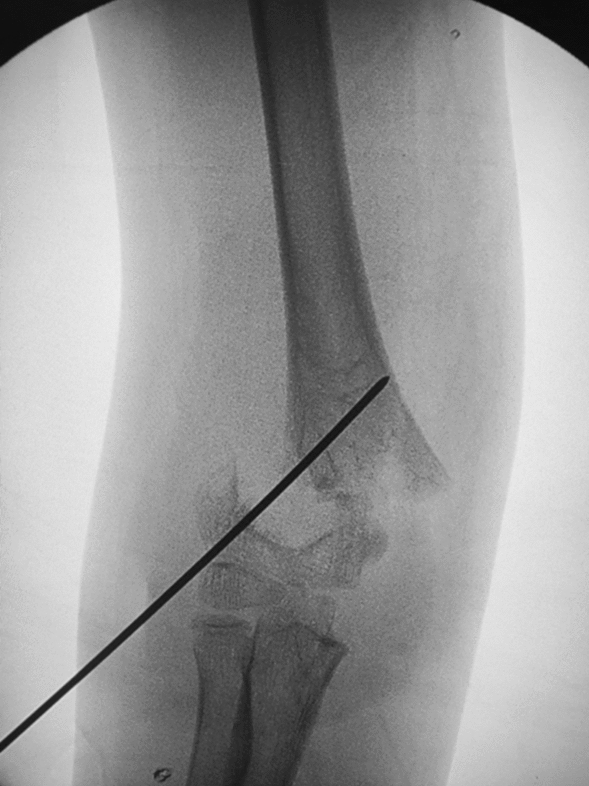
Intraoperative fluoroscopy in the anterior-posterior view shows the 'de-sharpened' Kirschner wire drilled into the distal end of the fracture block about at a 45° angle

**Fig. 5:**
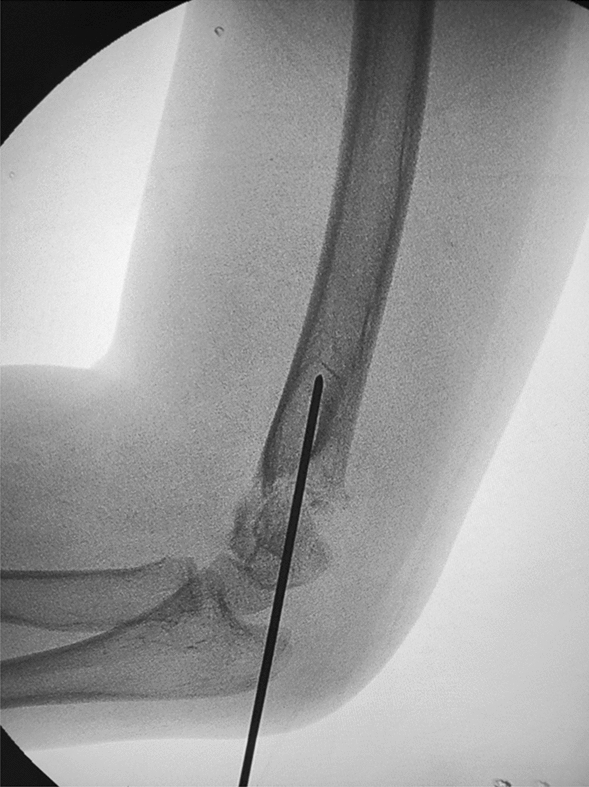
Intraoperative fluoroscopy in the lateral view shows the 'de-sharpened' Kirschner wire drilled parallel to the axis of the humeral shaft

**Fig. 6:**
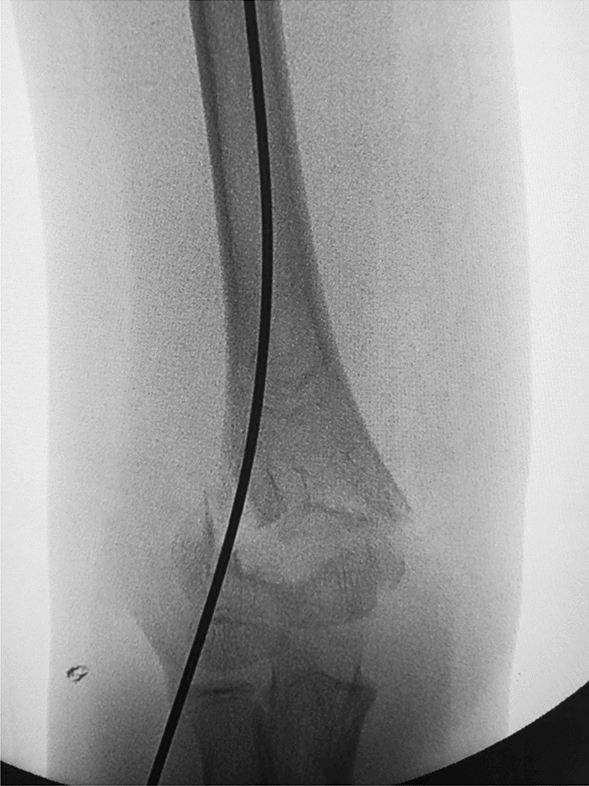
Intraoperative fluoroscopy in the anterior-posterior view shows the 'de-sharpened' Kirschner wire drilled into the medullary cavity, improving the outward displacement deformity

**Fig. 7:**
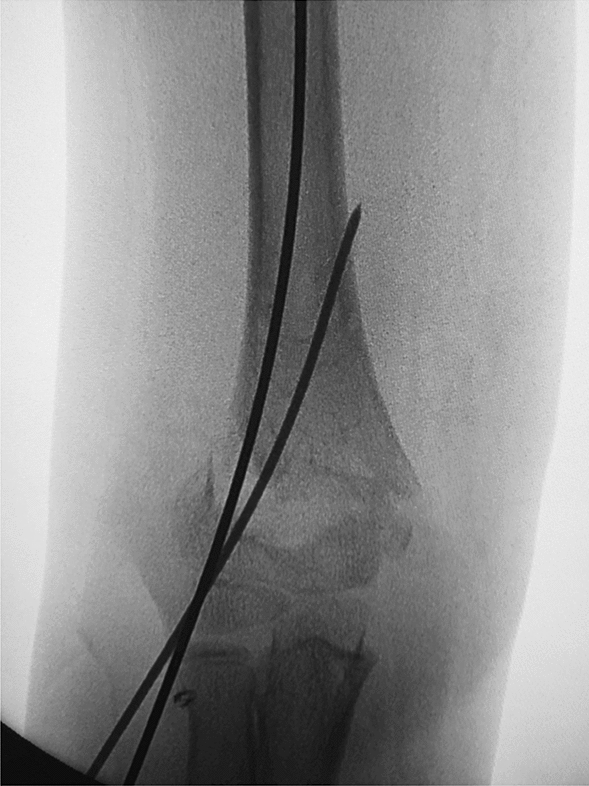
Intraoperative fluoroscopy in the anterior-posterior view shows another Kirschner wire passing through the fracture line and the opposite cortical bone, achieving satisfactory fracture reduction

**Fig. 8:**
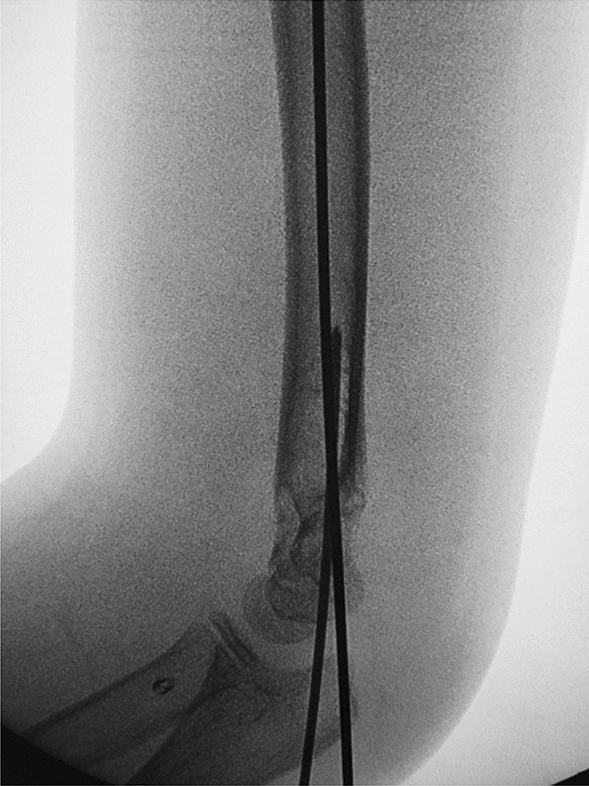
Intraoperative fluoroscopy in the lateral view shows satisfactory fracture reduction

**Fig. 9:**
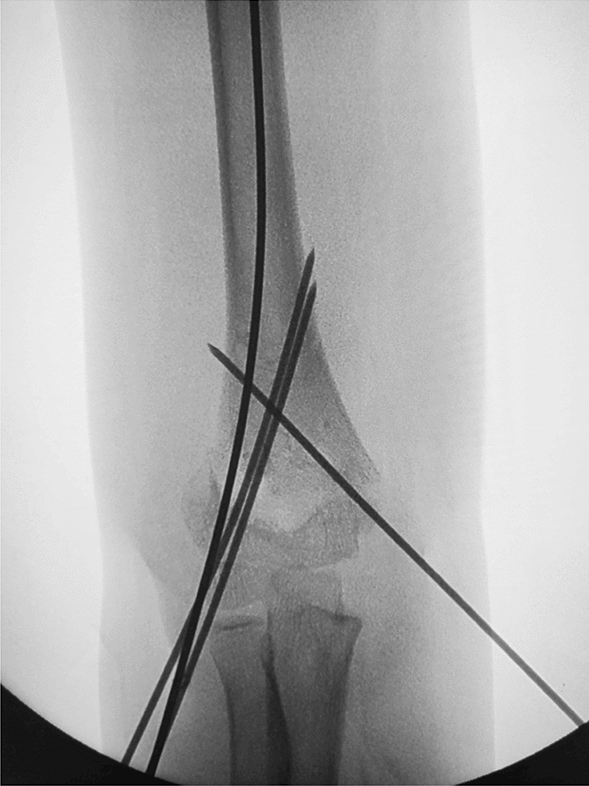
Intraoperative fluoroscopy in the anterior-posterior view shows cross-pinning with Kirschner wires on the medial and lateral sides, ensuring satisfactory fracture reduction

**Fig. 10:**
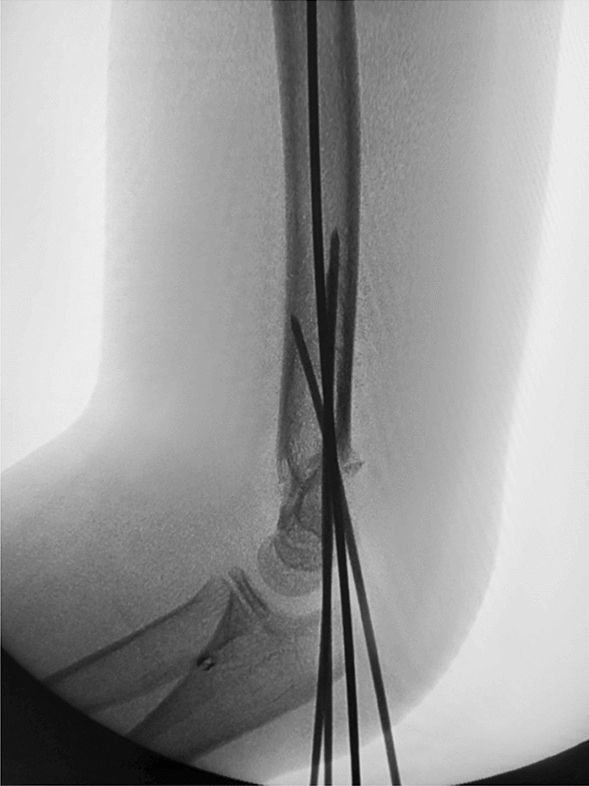
Intraoperative fluoroscopy in the lateral view shows satisfactory fracture reduction

Depending on the age of the child, we choose a 2.0-mm or 1.5-mm Kirschner wire and ground its tip with a file. We define this step as the de-sharpened of the Kirschner wire (Fig. 1). Further, we installed this Kirschner wire on the electric drill for use during the operation. The operator and assistant continued to traction the fracture; using the stability of the posterior periosteal hinge of the humerus, the operator pushed the distal fracture block from back to front with his thumb, reduced the sagittal displacement, and confirmed it by X-ray fluoroscopy. The operator then drilled the previously de-sharpened Kirschner wire into the lateral condyle of the humerus and stopped when the top of the Kirschner wire touched the medial bone cortex at a 45° angle with the axis of the humeral shaft. The Kirschner wire was pushed back to 2 mm, and then the operator pushed the distal fracture block medially to correct the lateral displacement, and slowly slid the top of the Kirschner wire into the bone marrow cavity of the humerus. After the Kirschner wire entered the medullary cavity, the depth of the Kirschner wire was adjusted according to the coronal displacement. The deeper the Kirschner needle enters, the stronger the power to correct the outward displacement. When the coronal displacement of the fracture was satisfactorily corrected, the traction of the fracture was continued and the rotational displacement was corrected by supination of the upper arm. It is good to determine the position of the fracture in all directions. The operator drilled two Kirschner wires from the lateral condyle of the humerus, and then drilled the other Kirschner wire at the medial epicondyle of the humerus. The internal and external Kirschner wires were cross-fixed. When drilling the medial wire, the operator placed the elbow joint on the medial side and in an incompletely extended position. At the same time, the operator touched the medial epicondylar with his thumb and blocked the posterior ulnar nerve. (Figs. 2, 3, 4, 5, 6, 7, 8, 9, 10).

#### Control group

The operator and assistant continued to traction the fracture as described above. First, the operator corrected the lateral displacement of the fracture and then extremely supinated the forearm to correct the rotational displacement. Finally, when the elbow was in gradual flexion, the operator used his thumb to push the distal fracture block from back to front to correct the sagittal deformity. These steps required assistance from the surgical assistant, and the fracture was under traction at all times. After the above reduction operation, the operator wound the bandage in the extreme flexion position to maintain the position after reduction. If the location of the fracture was found to be poor after fluoroscopy, the above reduction process was repeated. It is good to determine the position of the fracture in all directions. The operator drilled two Kirschner wires from the lateral condyle of the humerus, and then drilled the other Kirschner wire at the medial epicondyle of the humerus. The internal and external Kirschner wires were cross-fixed.

The operator first bent the tail of the Kirschner wire and left it outside the skin, and then wrapped the nail tail with a long strip of sterile gauze to protect it. In the final step, the operator fixed the patient’s upper limb in the flexion elbow position using full-length plaster.

### Postoperative management

After the operation, the patients were given symptomatic treatment such as hemostasis and detumescence, and attention was focused on any swelling of the extremities, the peripheral blood circulation, and sensory activity of the fingers. When swelling of the affected limb significantly improved and there was no exudation of the wire during the dressing change, the patient was discharged from the hospital. After discharge, the patient’s parents were given clear instructions on how to change the dressing regularly; the patient was advised to carry out functional exercises such as fist clenching. One month after the operation, the patient was followed-up at the outpatient clinic for radiographic assessment. For example, after adequate callus growth, the Kirschner wire was pulled out and the patient instructed to carry out elbow flexion and extension. The fracture healing and elbow joint function of the patients were evaluated 3 months and 6 months after the operation.

The operation time, number of intraoperative fluoroscopies, and clinical healing time of the fracture were compared between the two groups. The Baumann angle and carrying angle on the postoperative radiographs of the two groups were measured, and changes in the Baumann angle were compared. The Flynn function score of the elbow joint at 6 months after the operation was evaluated and compared.

### Statistical analysis

Statistical analysis was performed using SPSS23.0 software (IBM Corporation, Armonk, NY, USA). Normal distributed measurement data were expressed as *x* ± *s*, and the comparison between groups was tested by independent sample *t*-test. The count data were expressed as frequency and percentage, and the comparison between groups was tested by the chi-squared test. *P* < 0.05 was considered to indicate statistically significant differences.

## Results

The surgical operating time was 32.88 ± 3.69 min in the experimental group and 45.56 ± 10.13 min in the control group (*P* < 0.05). The number of intraoperative fluoroscopies was 20.62 ± 5.41 in the experimental group and 32.48 ± 8.20 in the control group (*P* < 0.05). The clinical healing time was 33.04 ± 5.12 days in the experimental group and 33.81 ± 4.72 days in the control group (*P* > 0.05). The change of Baumann angle after the operation in the experimental group was 3.24 ± 0.84°and that in the control group was 5.75 ± 1.25°, (*P* < 0.05). The postoperative effect was evaluated according to the elbow Flynn score: the excellent and good rate was 98.00% in the experimental group and 92.86% in the control group. The difference was not statistically significant (*P* > 0.5) (Table 2). There were no complications such as osteomyelitis, poor fracture healing, osteofascial compartment syndrome, iatrogenic vascular and nerve injury, and myositis ossificans in both groups.

**Table 2:** Comparison of operation time, frequency of intraoperative fluoroscopy, fracture healing time and Baumann Angle difference between the affected side and the healthy side at 3 and 6 months after surgery between two groups of Gartland type III posterolateral displaced supracondylar fracture of the humerus in children (*n*) ($\overline{x}$ ± *s*)

Groups	*n*	Operation time (min)	Frequency of intraoperative fluoroscopy (times)	Fracture healing time (d)	Difference between the affected side and the healthy side	
					3 months after surgery	6 months after surgery
Study group	50	32.9 ± 3.7	20.6 ± 5.4	33.0 ± 5.1	3.2 ± 0.8	2.3 ± 0.6
Control group	56	45.6 ± 10.1	32.5 ± 8.2	33.8 ± 4.7	6.0 ± 2.1	5.8 ± 1.3
${x}^{2}$/*t* Value		− 8.75	− 8.89	− 0.80	− 8.37	− 16.70
*P* value		< 0.001	< 0.001	0.428	< 0.001	< 0.001

Study group were treated with “de-sharpened” intramedullary elastic reduction with Kirschner wire, Control group were treated with traditional three-dimensional manipulation

## Discussion

Supracondylar fractures of the humerus in children can be classified into the extension type and flexion type according to the injury mechanism, although the extension type is more common than the flexion type. On the basis of the Gartland classification, Wilkins [2] classified type III fractures into posteromedial displacement type IIIa and posterolateral displacement type IIIb, and the incidence of the latter was lower than that of the former. For Gartland type III fractures, closed reduction and percutaneous Kirschner wire internal fixation are adopted to ensure less trauma and good joint function, which most research scholars and clinicians agree on.

Ondina et al. [7] studied 362 patients and reported that the posterolateral displacement of Gartland type III fracture was the influencing factor of failure of closed reduction and then open reduction. Novais et al. [8] conducted a study on 1592 patients and also suggested that in completely displaced humeral supracondylar fractures, posterolaterally displaced humeral supracondylar fractures are the same as flexion-type humeral supracondylar fractures, and the failure rate of closed reduction is higher than other types of fractures. This result is related to different injury mechanisms of posterolateral displaced fractures. In posterolateral displaced supracondylar fracture of the humerus, the trauma spreads from the palm to the anterior medial side of the distal end of the humerus, and the distal fragment moves posterolaterally. The lateral cortex of the humerus collapses due to compression, the medial periosteum of the humerus is completely broken, the distal fragment is displaced laterally, and the medial end of the proximal fragment rotates forward, which is a strong rotation force. The traditional three-dimensional reduction method is used to fight against traction, internal push and external pull, pronation or supination, and finally to push the fragment and bend the elbow from back to front. Most Gartland type III supracondylar fractures of the humerus, especially the posterior medial displaced supracondylar fracture, can be operated using this method. Maintaining pronation flexion elbow position can maintain the reduction of the fracture. However, some difficulties are often encountered in the treatment of posterolateral displaced supracondylar fracture of the humerus. The author found some problems in the treatment of posterolateral displaced supracondylar fracture of the humerus.

First, when the elbow joint is placed in the flexion position to maintain fracture stability, the stability can be easily lost, and the lateral displacement cannot be corrected; even the proximal medial rotation displacement of the fracture becomes relatively difficult to correct. At this point, the assistant is required to keep the forearm in the supination position, push the fragment of the distal fracture to the medial humerus, and maintain this position all the time. Second, when the medial Kirschner wire is placed, the patient's elbow joint is not fully extended and the medial side of the elbow joint faces upward, and the corrected rotation displacement of the medial humerus will reappear. Third, because of the poor stability of the fracture, the extent of Kirschner wire drilling and bone cortex injury increases, and the number of intraoperative fluoroscopies increases, in turn increasing the risk of radiation. For posterolateral displaced supracondylar fracture of the humerus, the traditional three-dimensional reduction method needs repeated reduction, which will aggravate the injury to the soft tissue and bone of the elbow, may affect the elbow joint function. In this study, the technique of intramedullary elastic reduction of the “de-sharpened” Kirschner wire for the treatment of Gartland type III posterolateral displaced supracondylar fracture of the humerus in children can shorten the operation time, reduce the reduction times, and correct the Baumann angle more effectively.

The technique of intramedullary elastic reduction of the “de-sharpened” Kirschner wire is performed to correct the shortening deformity through full traction. Relying on the thick periosteum on the posterior side of the humerus, the operator placed the elbow in the flexion position to correct the sagittal deformity. Finally, a de-sharpened Kirschner wire was drilled into the medullary cavity of the humerus from the lateral condyle of the humerus, and the lateral displacement was reduced by the elastic force of the Kirschner wire. At the same time, the forearm was placed in the supination position to correct the rotation deformity. Its advantages are as detailed below:

First, the technique of intramedullary elastic reduction of the “de-sharpened Kirschner wire is more reasonable.

Many scholars have reported a variety of techniques for auxiliary reduction with Kirschner wires when it is difficult for them to reduce supracondylar fractures of the humerus [9–12]. Most scholars use the joystick technology of Kirschner wire to obtain good reduction by controlling the distal and proximal end of the fracture. Dong et al. [13] and Basaran et al. [11] drilled a 2.0-mm Kirschner wire vertically on the distal fragment as a joystick to control the distal fragment, which achieved closed reduction of multidirectional unstable supracondylar fracture of the humerus and shorter operation duration. Other scholars use Kirschner wires as levers to pry fracture fragments and correct sagittal and rotational deformities, similar to the Kapandji techniques [14]. Wei et al. [12] reported the application of Kirschner wire joystick technique in the treatment of humeral supracondylar fractures in children. A Kirschner wire was drilled into the humeral bone marrow cavity from the ulnar olecranon to replace the posterior periosteal hinge for reduction. In this study, the Kirschner wire was also used for auxiliary reduction of fracture. The difference is that after the Kirschner wire is de-sharpened, the entry point is closer to the edge of the lateral condyle of the humerus to avoid damage to the olecranon fossa of the ulna; moreover, de-sharpening also ensures that the angle between the Kirschner wire and the axis of the humeral shaft can be larger. In addition, once the Kirschner wire tip loses its sharpness, it can easily slide into the medullary cavity along the bone cortex, which greatly reduces the difficulty of the Kirschner wire drilling into the medullary cavity. With the help of the elastic force of the Kirschner wire, the coronal deformity can be corrected and can act as a posterior and lateral hinge to temporarily stabilize the fracture. At the same time, because the Kirschner wire does not pass through the medial bone cortex of the humerus, it is easier to correct the rotation deformity later, and the Kirschner wire is not at risk of breaking. In this study, the operation time and the frequency of intraoperative fluoroscopy in the experimental group were 32.9 ± 3.7 min and 20.6 ± 5.4 times, respectively, which were much lower than those in the control group (32.5 ± 8.2 min and 32.5 ± 8.2 times, respectively). There are few studies on posterolateral supracondylar fracture of the humerus. Lim et al. [15] retrospectively analyzed 494 patients with supracondylar fracture of the humerus and found that the average operation time of 21 cases of posterolateral displacement was 37.86 min, which was longer than that of the study group in this study. In the future, with increasing proficiency of this surgical technique, the operation time will be further shortened.

Second, this technique is more accurate and effective in correcting coronal and rotational deformities of supracondylar fractures of the humerus.

Prusick et al. [16] and Smuin et al. [17] emphasized that when the operator performed manual reduction of posterolateral supracondylar fracture of the humerus, the patient's forearm was placed in supination position, and the elbow joint was placed in flexion position to correct the deformity by relying on the posterior and lateral hinges of the fracture. However, if the rotation deformity is very serious, it is difficult to correct it solely by manual reduction. Basaran et al. [11] proposed a new joystick technology. During manual reduction, a 1.2-mm Kirschner wire was drilled from the lateral condyle of the humerus and passed through the medial bone cortex of the humerus to temporarily fix the fracture. Finally, because the 1.2-mm Kirschner wire was easy to bend, the residual coronal deformities and rotation deformities were corrected by prying the fragment or by manual reduction. However, the author of this article believes that after correcting the rotation deformity, the 1.2-mm Kirschner wire may break, or it may be difficult to pull out because it becomes bent. In this study, the coronal deformity is corrected by the elastic force of the Kirschner wire in the medullary cavity. The deeper the Kirschner wire is drilled into the medullary cavity, the greater the elastic force is. The coronal deformity can be corrected by adjusting the depth of the drilled Kirschner wire. Because the Kirschner wire is in the medullary cavity, when the residual rotation deformity is corrected by manual reduction or pry reduction, the Kirschner wire will not bend and can be pulled out easily after the operation. In this study, the changes in Baumann angle in the experimental group were lower than those in the control group at 3 and 6 months after operation, which confirmed that this method could better correct coronal and rotation deformities.

Third, this method can obtain a better surgical position during the operation, and the stability of the fracture will not be lost during C-arm fluoroscopy.

In traditional manual reduction, anteroposterior radiographs are usually obtained in extreme flexion fluoroscopy, but it is not accurate to judge coronal deformities because of the interference of the ulna and radius. When obtaining lateral radiographs, the operator usually moves and rotates the elbow joint relative to the rest position of the C-arm receiver, which may aggravate the injury. Leitch et al. [18] pointed out that in the treatment of supracondylar fracture of the humerus, rotating the patient’s arm to obtain lateral radiographs will lead to the loss of fracture stability. Kamath et al. [19] proposed that it is ideal to obtain biplanar or multiplanar radiographs by rotating the C-shaped arm rather than rotating the injured limb. In this study, the Kirschner wire was drilled into the humeral bone marrow cavity from the lateral condyle of the humerus and played a temporary role in fixing the fracture, and the elbow joint could be completely straightened on the C-arm receiver to obtain anteroposterior radiographs. This method can not only avoid the overlapping influence of the ulna and radius during elbow flexion fluoroscopy but also rotate the affected limb and place it on the C-arm receiver to take lateral radiographs to judge the correction of sagittal deformities of fractures.

Fourth, the follow-up of this method showed that the prognostic function recovered well and there were only few complications.

Osateerakun et al. [20] found that repeatedly adjusting the distribution of Kirschner wires and repeated manual reduction can lead to complications such as wire tract infection, Kirschner wires loosening, vascular and nerve injury, and myositis ossificans. The technique proposed in this article is aimed at correcting the sagittal deformity first, then drilling the de-sharpened Kirschner wire into the medullary cavity of the humerus from the lateral condyle of the humerus as temporary fixation, and finally proceeding to the next step. This method can reduce the number of manual reductions, avoid soft tissue injury caused by repeated manual reduction, and avoid the occurrence of myositis ossificans in the later stage that can result in elbow joint dysfunction. At the same time, this method does not have to try to drill into the Kirschner wire repeatedly, thus reducing the risk of nail-hole infection, osteomyelitis, and iatrogenic vascular and nerve injury. At the last follow-up in this study, the Flynn score of the elbow joint function for the excellent and good rate of the experimental group was 98.0% (49 to 50) and that of the control group was 92.9% (52 to 56). There were no complications such as osteomyelitis, poor fracture healing, osteofascial compartment syndrome, iatrogenic vascular and nerve injury, and myositis ossificans in either group.

There are some limitations to the study. No biomechanical test was carried out, the number of cases in each group was less, and the observation index was also small. Therefore, it is necessary to continue to study the effect of the newly proposed technique in a larger sample cohort and using relevant biomechanical tests to further validate the therapeutic effect of this technique.

## Conclusion

For Gartland type III posterolateral displaced supracondylar fracture of the humerus in children, the technique of intramedullary elastic reduction of the “de-sharpened” Kirschner wire has a good therapeutic effect, which is similar to that of the traditional three-dimensional reduction. However, the former can shorten the operation time, reduce the frequency of intraoperative fluoroscopy, and better correct coronal and rotational deformities than the latter.

## Data Availability

We confirm that the data used in this study are available upon request. Researchers interested in accessing the data for further analysis or verification may contact Yudong Lin at ahpolinyudong@163.com for inquiries.
